# Case report: A combination of mirror therapy and magnetic stimulation to the sacral plexus relieved phantom limb pain in a patient

**DOI:** 10.3389/fnins.2023.1187486

**Published:** 2023-05-26

**Authors:** Chunchu Deng, Qian Li

**Affiliations:** Department of Rehabilitation, Tongji Hospital, Tongji Medical College, Huazhong University of Science and Technology, Wuhan, China

**Keywords:** case report, mirror therapy, magnetic stimulation, phantom limb pain, effective treatment

## Abstract

Phantom limb pain (PLP) is a common sequela of amputation, experienced by 50-80% of amputees. Oral analgesics as the first-line therapy have limited effects. Since PLP usually affects activities of daily living and the psychological conditions of patients, effective treatments are imperatively needed. In this case study, a 49-year-old man was admitted to our hospital because of uncontrollable paroxysmal pain in his missing and residual leg. Due to severe injuries in a truck accident, the right lower limb of the patient was surgically amputated ~5 years ago. Around 1 month after amputation, he felt pain in his lost leg and PLP was diagnosed. Then, he started taking oral analgesics, but the pain still occurred. After admission on July 9, 2022, the patient received treatments of mirror therapy and magnetic stimulation to the sacral plexus. 1-month treatments reduced the frequency and intensity of pain in the phantom limb and the stump, without any adverse events. Analysis of high-resolution three-dimensional T1-weighted brain volume images at the end of 2-month treatments showed alterations in the thickness of cortex regions related to pain processing, compared to that before treatment. This case study gives us hints that one or both interventions of mirror therapy and sacral plexus magnetic stimulation effectively relieved PLP and stump limb pain. These non-invasive, low-cost and easily conducted treatments could be good options for PLP. But randomized controlled trials with a large number of cases are required to confirm their efficacy and safety.

## 1. Introduction

Phantom limb pain (PLP) is defined as a painful perception of a limb that has been already amputated. There were 1.6 million amputees in the United States in 2005, and the number was estimated to increase to 3.6 million by the year 2050 (Ziegler-Graham et al., 2008). PLP occurs within 1–3 months after amputation in ~49%−93% of amputees (Kooijman et al., 2000; Kaur and Guan, 2018). It usually manifests as stabbing, cramping, numbness or burning pain (Fuchs et al., 2018), which can affect amputees' ability in daily activities and their psychological states (Ahmed et al., 2017; Aternali and Katz, 2019).

A variety of pharmacological and non-pharmacological treatments have been reported to manage PLP (Hall and Eldabe, 2018; Hyung and Wiseman-Hakes, 2022). However, it is difficult to judge from the available data which methods are more effective, and a widely accepted consensus hasn't been reached (Hall and Eldabe, 2018; Hyung and Wiseman-Hakes, 2022). Mirror therapy (MT) and magnetic stimulation are non-pharmacological interventions for PLP. During MT, a mirror is positioned midsagittally in front of the patient to create a reflection of an unaffected body part as if it were the affected part (Thieme et al., 2018). Magnetic stimulation is a method of delivering a magnetic field to penetrate tissues and stimulate nerves (Brodak et al., 1993). High-frequency (>5 Hz) magnetic stimulation has an excitatory influence on the cortex, whereas low-frequency (1 Hz or lower) exerts inhibitory effects (Cruccu et al., 2016; Kanjanapanang and Chang, 2023). Repetitive transcranial magnetic stimulation (rTMS) at a high frequency is usually used for PLP (Nardone et al., 2019). Repetitive magnetic stimulation of sacral roots was applied to evaluate the function of the motor and sensory pathways (Brodak et al., 1993) and to shortly relieve sciatica and pudendal neuralgia (Sato and Nagai, 2002). But whether magnetic stimulation to peripheral nerves like the sacral plexus could help manage PLP remains enigmatic (Beaulieu and Schneider, 2015).

The central and peripheral nervous systems and psychological aspects are involved in the pathogenesis of PLP (Hyung and Wiseman-Hakes, 2022), indicating that a combination of interventions that interfere with different affected systems could be a promising strategy. Here, we report the positive clinical effects of MT combined with sacral plexus magnetic stimulation on a patient with trauma-related PLP and residual limb pain (RLP) that could not be controlled with oral analgesics.

## 2. Case description

On March 8, 2017, a 49-year-old man was hit by a truck, with severe injuries to multiple body regions. Upon admission to a hospital, his right lower limb was surgically amputated based on clinical examinations and evaluation. In addition, he underwent left calf debridement, vacuum sealing drainage, abdominal exploration and repair, suprapubic cystostomy, adipose tissue transplantation, skin stretching, skin grafting after debridement on the right hip, and urethral reconstruction from March to May 2017. All the surgical treatments were successful and his general condition became stable. Around 1 month after amputation, the patient felt pain in his missing leg and PLP was diagnosed. Then, he started taking oral analgesics. But the pain in the missing limb still occurred and affected his life quality.

**Figure 1:**
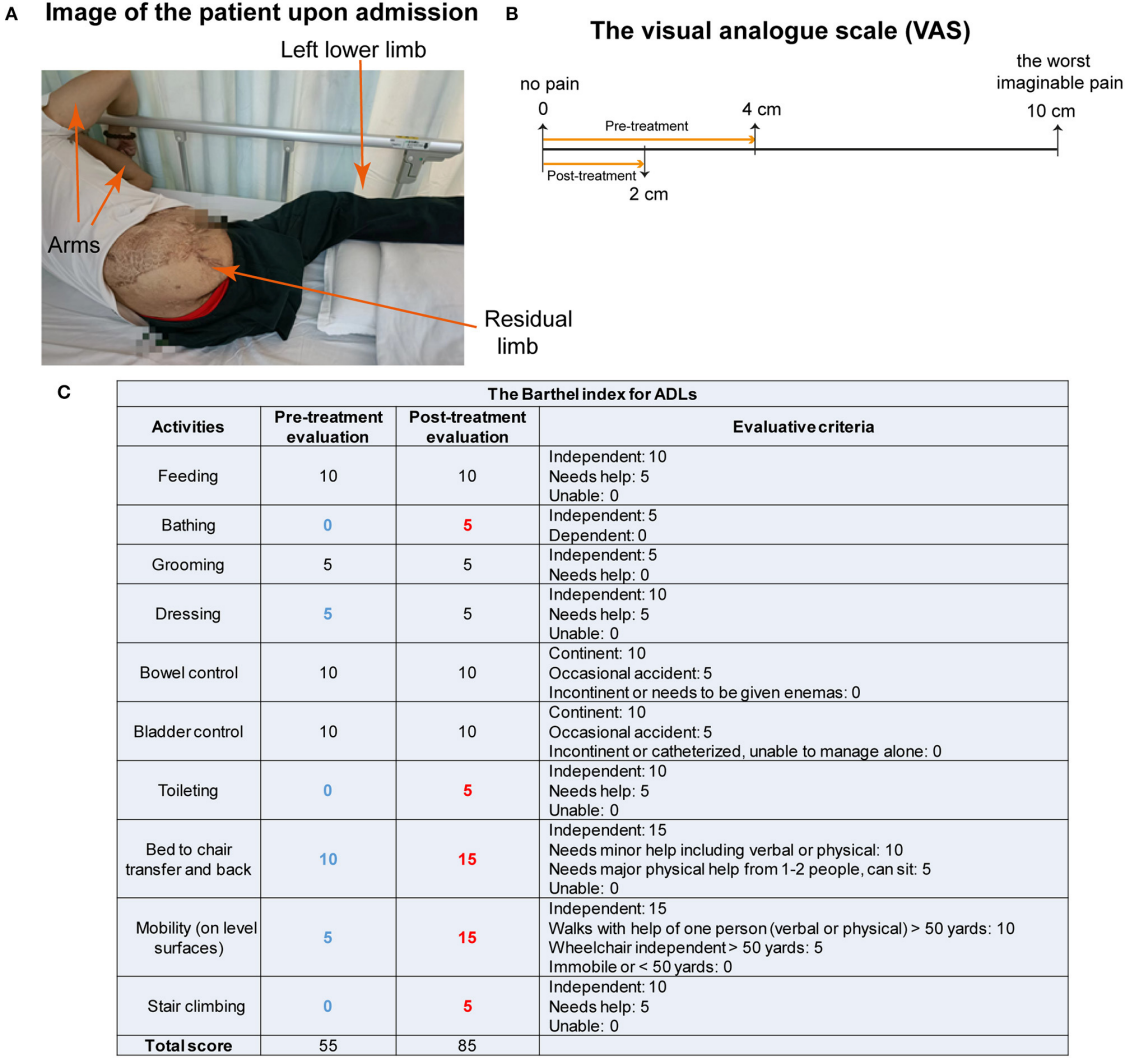
Assessments of the patient's condition, VAS and ADLs. **(A)** The patient's condition upon admission. **(B)** Assessment of the VAS shows scores of 4 cm before treatment and 2 cm after 1-month treatment of mirror therapy and magnetic stimulation. **(C)** Evaluation of the Barthel index for ADLs demonstrates the ability in fundamental skills pre-treatment and after 1-month treatment.

**Figure 2:**
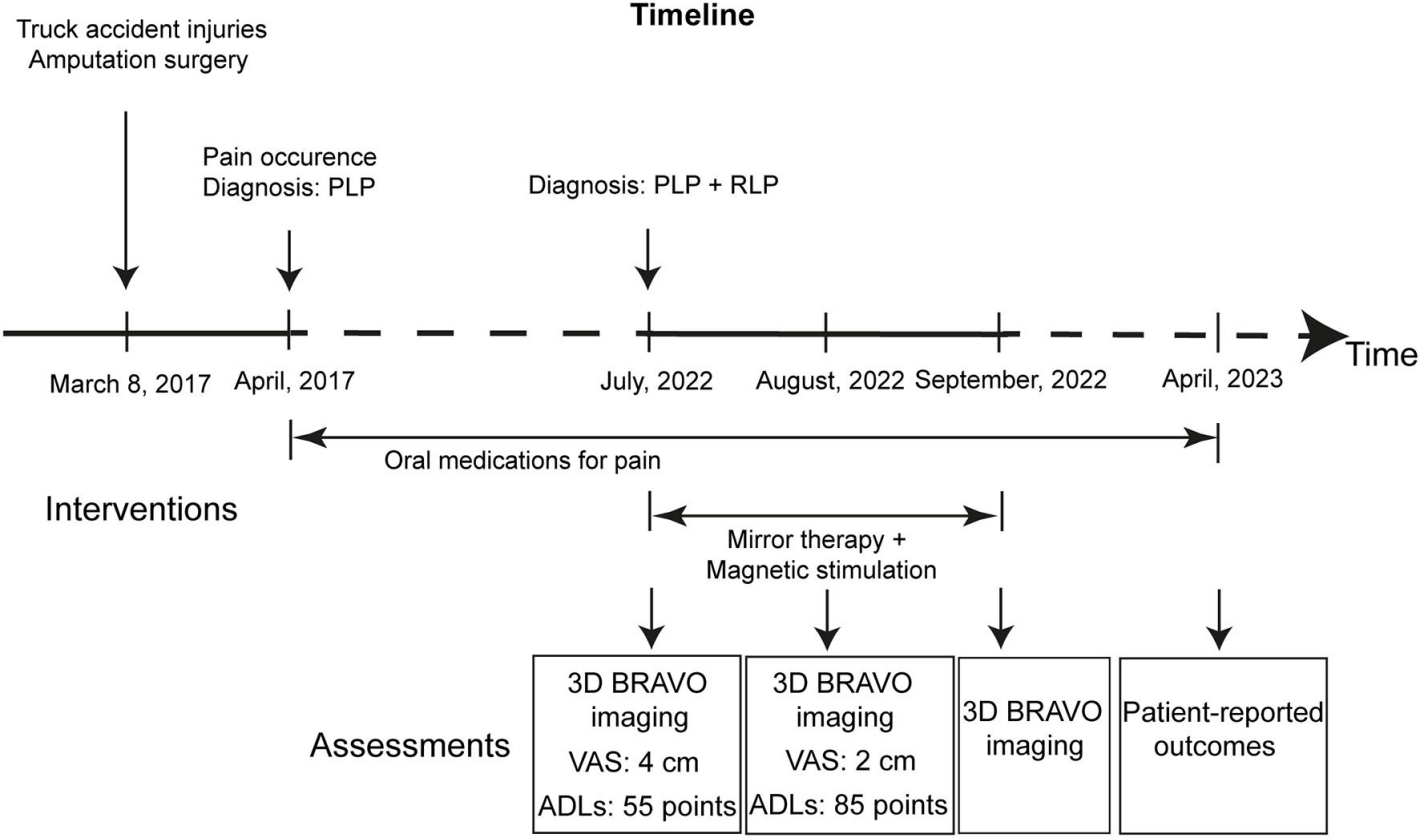
Timeline showing the relevant events in the case.

On July 9, 2022, the patient was admitted to our hospital because of pain. He also complained of a bad spiritual state. Upon admission, we checked the residual limb, and found it in good condition, without swelling or fever (Figure 1A). On the same day, pain intensity was assessed using the visual analog scale (VAS) that contains a 10 cm line with statements anchored on the left (no pain) and on the right (the worst imaginable pain) (Delgado et al., 2018). The patient was asked to draw a line vertically to the 10 cm VAS line at the point that represented his pain intensity. After this, a ruler was used to measure the distance between the patient's mark and the “no pain” on the VAS line. The score varies from 0 to 10 cm. The higher score indicates greater pain intensity. The pre-treatment score of the patient was 4 cm (Figure 1B). Additionally, the ability in activities of daily living (ADLs) was measured using the Barthel index for ADLs (Mahoney and Barthel, 1965; Shah et al., 1989). Based on previous publications and MDCalc calculator (Mahoney and Barthel, 1965; Shah et al., 1989; Quinn et al., 2011; Elovic and Pourmand, 2019), ADLs in the assessment contain feeding, bathing, grooming, dressing, bowel control, bladder control, toilet use, transfers from bed to chair and back, mobility on level surfaces, and stair climbing. The total score is 100 points, with a higher score indicating more independence. The pre-treatment score of ADLs was 55 points, with 0 points for bathing, toilet use, and stair climbing, and with decreased points for dressing, transfers, and mobility on level surfaces (Figure 1C). The patient had type 2 diabetes mellitus. Oral vildagliptin and metformin controlled the blood glucose at normal values. There was no special family history, no psycho-social history, and no use of psychoactive medications. The timeline (Figure 2) shows the relevant events in this case from amputation to the follow-up.

### 2.1. Diagnostic assessment, therapeutic interventions, follow-up and outcomes

#### 2.1.1. Diagnostic assessment

Based on the medical history and clinical symptoms, PLP and RLP were diagnosed. The pain occurred around 1 month after amputation, mainly in the non-existing limb and, to a lesser extent, in the residual limb. Paroxysmal pain was induced by emotional and climate changes, fatigue or other diseases. Sometimes, it occurred without any stimuli. The patient could not describe the characteristics of the pain. The duration of pain attacks varied from seconds to hours. Gently touching the stump could trigger pain that radiated to the distal part of the non-existing limb. PLP, localized in the removed part of the limb, manifests as acute and chronic pain, while RLP occurs more acutely after surgery (Modest et al., 2020). PLP usually presents as stabbing, cramping, numbness, burning or shooting pain (Hogan et al., 2022). RLP may provoke PLP (Flor et al., 1995). Differentiated from PLP, first, the affected range of RLP is relatively diffuse, which can affect the entire stump and radiate to other parts of the body. Second, RLP is described as tingling, burning or jumping pain. Third, severe pain and obvious tender points occur when the stump limb is touched. The mechanisms of PLP are complex, with altered peripheral nerve signals and abnormal reorganization of the central nervous system, while the main cause of RLP is the neuroma formation at the amputated site (List et al., 2021). It should not be neglected that RLP often co-exists with PLP (Hsu and Cohen, 2013).

#### 2.1.2. Therapeutic interventions

**Figure 3:**
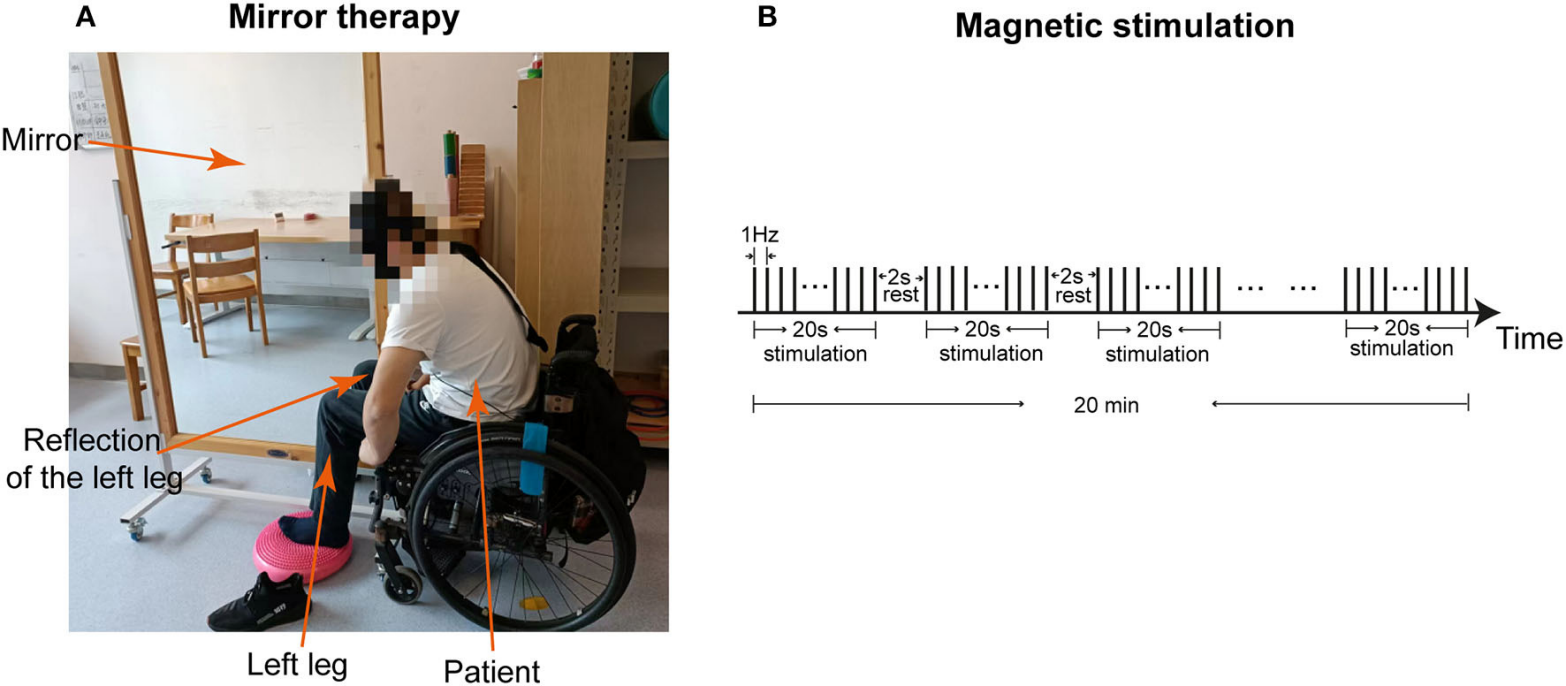
Interventions of mirror therapy and magnetic stimulation. **(A)** The image shows the patient who is receiving mirror therapy. **(B)** The graph demonstrates the program of magnetic stimulation at 1 Hz frequency for a total of 20 min, with 2 s rest between each 20 s stimulation.

After admission, interventions of MT and sacral plexus magnetic stimulation were provided to the patient, without stopping his previous medication, oral pregabalin, 75 milligrams (mg) 2 times a day. During MT, the mirror was placed vertically. The left intact lower limb of the patient was positioned on one side of the mirror, with the affected right side hidden on the other side, such that the patient could view the reflection of the left lower limb as if it were the right one (Figure 3A). While focusing on the reflection in the mirror and imagining that he could control the right lower limb, the patient was asked to perform a 20-min movement of the joints and muscles of the lower limbs. For magnetic stimulation, the magnetic field stimulator (YRD CCY-1, Yiruide Inc., Wuhan, China) was used (Yan et al., 2017) to simulate the plexus at Sacrum 3 because the sacral plexus receives sensory information from the lower extremities (Neufeld et al., 2015). Stimulation at a low frequency of 1 Hz, which was supposed to have inhibitory effects, was applied for a total of 20 min, with 2-s rest between each 20-s pulse (Figure 3B). The patient received 20 min of MT and 20 min of sacral plexus magnetic stimulation per day, from Monday to Friday in a week, for a total of 1 month.

#### 2.1.3. Follow-up

After being discharged from the hospital in August 2022, the patient received the same interventions in outpatient clinics every Monday to Friday for another 1 month. At the end of this 1-month treatment in September, high-resolution three-dimensional (3D) T1-weighted brain volume (BRAVO) images were obtained to compare to images scanned pre-treatment in July and after the 1st month of treatment in August to check whether there were changes in cortex regions related to pain processing. We followed up with the patient in April 2023 and assessed the outcomes based on oral information from the patient.

### 2.2. Assessment of high-resolution 3D T1-weighted BRAVO images

**Figure 4:**
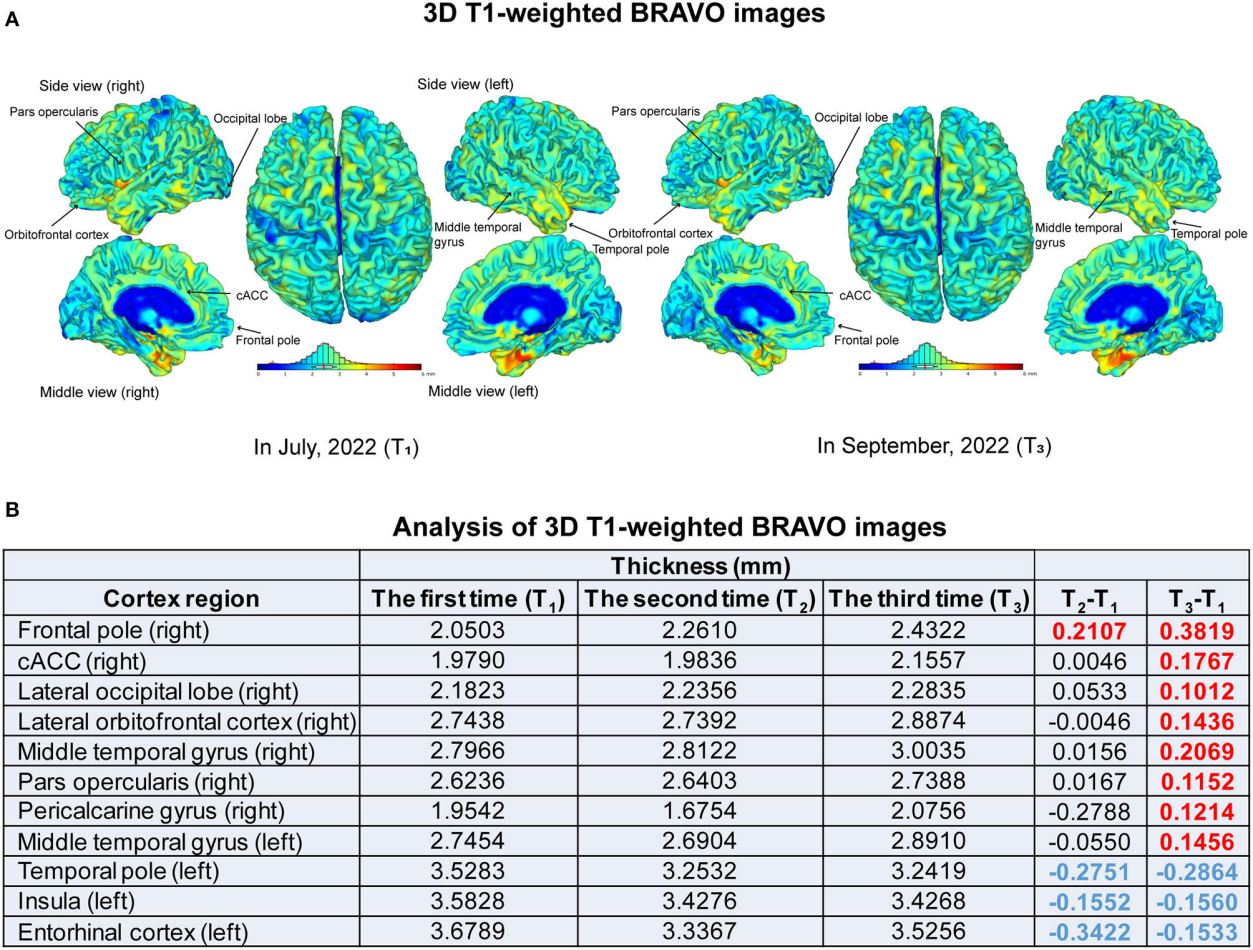
High-resolution 3D T1-weighted BRAVO images. **(A)** 3D BRAVO images before treatments (T_1_) and at the end of 2-month treatments (T_3_) of mirror therapy and sacral plexus magnetic stimulation. Different colors demonstrate different thicknesses, as shown with the color bar. The black arrows indicate regions with changed thickness. **(B)** Analysis of 3D T1-weighted BRAVO images obtained before treatment (T_1_) in July, at the end of 1-month treatment (T_2_) in August, and at the end of 2-month treatment (T_3_) in September 2022.

Using GE SIGNA Architect 3.0T (GE Healthcare, United States), high-resolution 3D T1-weighted BRAVO images (Figure 4A) were acquired with the following parameters: matrix size = 256 x 256 x 184, voxel size = 1 x 1 x 1 mm^3^, repetition time (TR) = 7.1 ms, echo time (TE) = 2.7 ms, flip angle (FA) = 12°. The raw images were processed with the *Computational Anatomy Toolbox* (CAT) toolbox (http://www.neuro.uni-jena.de/cat/) within the *Statistical Parametric Mappin*g (SPM) software package (http://www.fil.ion.ucl.ac.uk/spm/software/spm12/) using MATLAB to gather cortical morphological parameters (Gaser et al., 2022). After format conversion and reorientation, T1 images were entered into the preprocessing pipeline in CAT12 with the default parameters, including correction for bias-field inhomogeneities, tissue segmentation into gray matter, white matter, and cerebrospinal fluid, and normalization using the DARTEL algorithm. Estimation of CT and the central surface was performed in one step, based on the projection-based thickness (PBT) method (Dahnke et al., 2013). Before the statistical analysis, the CT maps were smoothed with a 15-mm FWHM Gaussian kernel.

### 2.3. Outcomes

The outcomes were the changes in the PLP intensity and ability of daily activities, indexed by the VAS and the Barthel index for ADLs from pre-treatment to the end of 1-month treatment (post-treatment) of MT and magnetic stimulation. The VAS was reduced to 2 cm from 4 cm, indicating a reduction in pain intensity (Figure 1B). The Barthel index for ADLs was increased to 85 points, with improved ability in bathing, toilet use, transfers, mobility on level surfaces, and stair climbing (Figure 1C). The frequency of pain attacks was reduced, assessed by clinicians and the patient. Furthermore, analysis of BRAVO images showed that the thicknesses of the frontal pole, caudal anterior cingulate cortex (cACC), lateral occipital lobe, lateral orbitofrontal cortex, pars opercularis, pericalcarine gyrus on the right side and bilateral middle temporal gyrus were increased more than 0.1 mm at the end of 2-month interventions (T_3_), compared to that pre-treatment (T_1_) (Figure 4B). The thickness increase in the right frontal pole occurred earlier and larger than that in other regions. On the contrary, thicknesses of the temporal pole, insula and entorhinal cortex on the left side were reduced after 1- and 2-month treatments (Figure 4B). These changes indicate that one or both interventions could induce changes in the brain cortex.

In the follow-up interview in April 2023, the patient said that he felt satisfied with the treatments and that his spiritual state and engagement in social life had improved. The pain still occurred, but it was very mild and not often. No adverse or unanticipated events due to the therapy were observed in this case.

## 3. Discussion

In this case, pharmacotherapy did not effectively relieve PLP in the last 5 years. The addition of MT and sacral plexus magnetic stimulation reduced the frequency and intensity of PLP and RLP. The ability of ADLs and life quality were improved. Furthermore, analysis of high-resolution 3D BRAVO images revealed thickness changes in cortex regions. Previous studies have shown that the frontal cortex plays important roles in pain processing such as pain perception and modulation (Lorenz et al., 2003; Schulz et al., 2015). The orbitofrontal cortex and ACC are functionally associated with the periaqueductal gray (PAG) that affects behavioral responses to harmful stimuli (Jones et al., 2003; Valet et al., 2004; Kong et al., 2010). The temporal pole, insula, entorhinal cortex, temporal gyrus, pars opercularis and pericalcarine gyrus are also related to pain perception (Liu et al., 2012; Caeyenberghs et al., 2017; Garcia-Larrea and Mauguière, 2018; Amin et al., 2021; Lin et al., 2022). These regions changed in our study, indicating that one or both interventions of MT and sacral plexus magnetic stimulation could induce changes in the brain cortex and affect pain perception. Additionally, the adherence and tolerability of the patient to these interventions were high due to their non-invasive and simple characteristics. However, the limitations of this case study should be addressed. First, it is difficult to evaluate the efficacy and safety of the combined interventions because the case number is not enough, and control groups are missing. Second, oral analgesics could interfere with the effects of combined interventions. Even though the oral medications did not relieve pain effectively, we could not exclude that the combined interventions might improve the efficacy of the analgesics. Third, the follow-up assessments should be improved.

Pharmacotherapy is considered the first-line treatment for PLP (Hall and Eldabe, 2018; Urits et al., 2019). There are a variety of pharmacological agents, such as non-steroidal anti-inflammatory drugs, anti-epileptic drugs, antidepressants, regenerative agents for the neuro-immuno-endocrine system and N-methyl-D-aspartate receptor antagonists (Hall and Eldabe, 2018; Urits et al., 2019). But oral analgesics alone are not able to manage the PLP in many cases (Hall and Eldabe, 2018), which is the same in this case. Other options for PLP are non-pharmacological treatments that involve rTMS, spinal cord stimulation (SCS), pulsed radiofrequency ablation (PRFA), and so on (West and Wu, 2010; Corbett et al., 2018; Urits et al., 2019). However, one treatment alone has a limited effect on the improvement of PLP and life quality (Corbett et al., 2018). Some of them are invasive and possibly cause permanent nerve damage (Corbett et al., 2018). Among non-invasive interventions, rTMS at a high frequency of 10 Hz or 20 Hz was used on the contralateral motor cortex (M1) for the treatment of PLP in many cases (Ahmed et al., 2011; Malavera et al., 2016). But the effective time varied from up to 15 days to months (Ahmed et al., 2011; Malavera et al., 2016). Other studies on rTMS of the cortex showed that it could relieve pain caused by spinal cord injury, stroke and so on (Khedr et al., 2005; Yilmaz et al., 2014). During the pathogenesis of PLP, synapses in the primary somatosensory and motor cortex are reorganized, and rTMS could alter the neuronal excitability and modulate affected synapse plasticity (Flor et al., 1995; Karl et al., 2001; King and Tang, 2022). Apart from the cerebral cortex, the spinal cord pathway is an important contributor to PLP pathogenesis, which was supported by the therapeutic effects of SCS (De Caridi and David, 2016; Aiyer et al., 2017). As the first relay site in pain pathways, the spinal cord receives the afferent information from the periphery and conveys it to the brain (D'Mello and Dickenson, 2008). Peripheral nerve injury causes continuous sensitization, synapse remodeling and weakened disinhibition of the spinal cord center, resulting in pain perception induced by non-nociceptive stimulators (D'Mello and Dickenson, 2008). Nerve injuries also induce the release of nociceptive chemicals, such as substance P, histamine and bradykinin (McHugh and McHugh, 2000). These chemicals can enhance the rate or transmission of nociceptive impulses (McHugh and McHugh, 2000). Therefore, we innovatively selected the peripheral nerves, namely the sacral plexus, as the site of the magnetic stimulation. The sacral plexus receives the afferent sensory messages from the lower extremities (Neufeld et al., 2015). A low frequency (1 Hz) of magnetic stimulation was supposed to inhibit the afferent signals of peripheral nociceptors, thus inhibiting the primary transmission of pain and consequently relieving pain. Surgical interventions of the lumbar sympathetic ganglion block or dorsal root entry zone lesions also can be used to block pain transmission (Zheng et al., 2009; Zhang et al., 2022). But, they are invasive and considered only when other treatments do not work effectively (Subedi and Grossberg, 2011).

Another non-invasive method in our study is MT which was first proposed in 1995 and initially used for PLP (Ramachandran et al., 1995). Data from electrophysiological experiments, fMRI and positron emission tomography have shown that cortical neurons that are involved in functions of the amputated limb can be altered during visualizing virtual movements of the missing limb in the mirror, thus reducing the PLP (Roux et al., 2003; Touzalin-Chretien et al., 2009; Diers et al., 2010). In our case study, analysis of high-resolution 3D BRAVO images revealed the increased thickness of the cortex regions that are responsible for pain perception and emotional management after treatments of MT and magnetic stimulation.

Conclusively, large samples, multi-center and high-quality randomized controlled trials are required to study various methods for PLP. Secondly, MT and sacral plexus magnetic stimulation at a low frequency might be a good combination because they target two major pathological mechanisms and they are non-invasive, low-cost and simple. Assessments of VAS, ADLs and 3D BRAVO images indicate the effective treatment of one or both interventions in our study. Thirdly, the neurobiological mechanisms underlying PLP need to be further investigated. This will help to develop multimodal or novel therapeutic strategies for PLP.

## 4. Patient perspective

I am happy that treatments of mirror therapy and sacral plexus magnetic stimulation helped me relieve pain in my right amputated leg. My ability in daily activities and my emotion have become better than before. These treatments are non-invasive and easily performed. I hope that more patients who suffer from PLP like me can also get a chance to these treatments and overcome the problems caused by amputation as many as possible.

The patient's perspective has given us positive feedback. Objective clinical assessments including the VAS, ADLs and MRI indicate the effectiveness of these treatments for PLP and RLP. However, large-scale randomized clinical trials are required for further confirmation. This will help more amputees with PLP in the world.

## Data availability statement

The original contributions presented in the study are included in the article/supplementary material, further inquiries can be directed to the corresponding author.

## Ethics statement

The studies involving human participants were reviewed and approved by the Ethics Committee of the Tongji Hospital, Tongji Medical College, Huazhong University of Science and Technology, Wuhan, China. The patients/participants provided their written informed consent to participate in this study. Written informed consent was obtained from the individual(s), and minor(s)' legal guardian/next of kin, for the publication of any potentially identifiable images or data included in this article. Written informed consent was obtained from the participant/patient(s) for the publication of this case report.

## Author contributions

QL contributed to the conception and design of the study and as well as the treatment and analysis of the data. CD contributed to the drafting of the manuscript. All authors contributed to the article and approved the submitted version.
